# Edge-preserving smoothing filter using fast M-estimation method with an automatic determination algorithm for basic width

**DOI:** 10.1038/s41598-023-32013-9

**Published:** 2023-04-04

**Authors:** Yudai Yamaguchi, Ichiro Yoshida, Yuki Kondo, Munetoshi Numada, Hiroyasu Koshimizu, Kaito Oshiro, Ryo Saito

**Affiliations:** 1 Graduate School of Science and Engineering, Research Institute of Precision Engineering and Data Science, Hosei University, Tokyo, Japan; 2 Faculty of Science and Engineering, Hosei University, Tokyo, Japan; 3 Faculty of Engineering, Chukyo University, Nagoya, Japan; 4 Faculty of Engineering, YYCSolution, Chukyo University, Nagoya, Japan; 5 Graduate School of Science and Engineering, Hosei University, Tokyo, Japan

**Keywords:** Computer science, Scientific data

## Abstract

Digital filtering is essential for digital imaging, image recognition, and super-resolution technology. For example, the presence of noise in images captured by digital cameras causes deterioration of the image quality and image recognition rate. In order to improve the image recognition rate, noise reduction and edge preservation must be performed during preprocessing. Noise is generally reduced using low-pass filters, such as the Gaussian filter. Although they reduce noise, such filters also have the properties of blurring edge. A strong edge blur reduces the accuracy of the feature detection in image recognition. Therefore, in our previous study, a fast M-estimation Gaussian filter for images (FMGFI) was proposed as an image filter that simultaneously achieves denoising and edge preservation. In the FMGFI, the setting of the optimal basic width of the 2nd order B-spline basis functions is important for achieving simultaneous denoising and edge preservation. In this method, the optimal basic width of the FMGFI was determined not only by manually setting the basic width but also by human judgment of the filtered images. Consequently, the inability to automatically determine the optimal basic width hindered efficient denoising during image processing Therefore, in this research, we develop and propose a method that can automatically determine the optimal basic width of the FMGFI. The previously proposed method calculates using the same basic width for all the pixels over the entire image; in contrast, the proposed method calculates using the basic width automatically determined for each pixel. The experiments confirmed that the method proposed in this study achieves higher denoising and edge preservation performance than the ones used in previous research. The results also showed that it has the highest denoising performance against salt-and-pepper noise as compared to other filters: non-local mean filter, Gaussian filter, median filter, bilateral filter, adaptive bilateral filter, and FMGFI. The experimental results for the Gaussian noise sowed that the proposed method has the same denoising and edge preservation performance as the other filters in visual evaluation. From the above, the proposed method is expected to contribute to efficient denoising and improvement of image quality by using it as a preprocessing.

## Introduction

Images captured using digital cameras include noise, which increases when the images are captured in dark areas^1^. Further, the image quality and image recognition rate decrease with the increase in noise^2–4^. High performance and large image sensors can reduce the rate of noise in an image; however, the rate cannot be reduced to zero. Therefore, noise reduction is necessary even for images captured by cameras with large image sensors and digital filtering is essential for digital imaging, image recognition, and super-resolution technology^5–7^ Noise is generally blurred and reduced using low-pass filters, such as the Gaussian filter (GF)^8–14^. Such filters have an advantage of reducing noise, however they also have a disadvantage of invariably blurring edge^2,10^. As the edge blur becomes stronger, the image quality decreases owing to the loss of resolution. In addition, if noise is included or the edges are blurred, the accuracy of feature detection for image recognition decreases^15,16^. Therefore, image filters with both denoising and edge preservation performances are required^17^.

Although the median filter (MF)^18–22^ has not been used frequently in recent years, it is capable of preserving edges. In recent years, the bilateral filter (BF)^23–25^, which is a normally distributed weighted GF, has been frequently used. This filter can preserve edges; however, it has a low denoising performance. In addition, it can reduce noise by adjusting the parameters, however, it blurs the edge. The non-local mean filter (NLMF)^26–29^ and adaptive bilateral filter (ABF)^30–33^, which are based on the BF, have high denoising performance against weak noise such as Gaussian noise^12,34–36^; however, in the case of strong noise such as salt-and-pepper noise, they perform poorly^37–39^. Furthermore, the NLMF is computationally expensive and requires a long processing time.

To solve the aforementioned problems, a fast M-estimation Gaussian filter for images (FMGFI) combining the GF and fast M-estimation methods was proposed in previous research^40^. The FMGFI achieves simultaneous denoising and edge preservation because it can remove the spike noise and preserve steps by utilizing the characteristics of fast M-estimation methods^41^. The key to achieving simultaneous denoising and edge preservation is to set the width of the 2nd order B-spline basis function (hereinafter referred to as the B. W.) to the optimum value in the FMGFI. In this previous research, the basic width of the FMGFI was set manually, and the filtered results had to be judged by a human. As a result, the method did not facilitate efficient denoising during image processing because the optimal basic width could not be automatically determined.

Therefore, in this study, we propose a method that can automatically determine the optimal basic width of the FMGFI. The proposed method is expected to have higher denoising and edge preservation performance than the ones used in previous studies because the basic width is calculated for each pixel. Therefore, the proposed method can improve the efficiency of denoising in image processing. In addition, the proposed method is expected to improve the image quality of images including noise and to be applied to preprocessing for image recognition and super-resolution technology. The recognition rate is also expected to be improved through the application of the proposed method to the preprocessing of image recognition. The remainder of this paper is organized as follows: we describe the fast M-estimation method in first section. The proposed method is described in next section. The verification experiments are presented in next section. Finally, the conclusions are presented in last section.

## Fast M-estimation method

**Figure 1:**
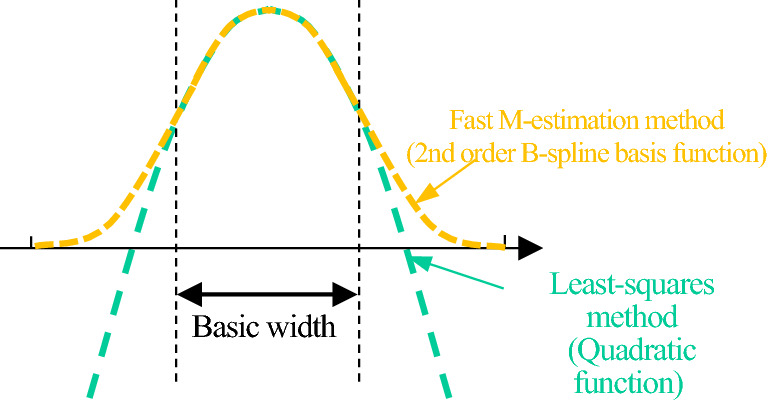
Loss function in the robust estimation.

A fast M-estimation method is one of the robust estimation methods. The fast M-estimation method uses a 2nd order B-spline basis function as the loss function. As shown in Fig. 1, the 2nd order B-spline basis function has the characteristic that weights in the basic width range around the center are equal to the quadratic function, and the weights outside the basic width range converge to zero. If the image does not include any noise, only the central base width range of the 2nd order B-spline basis function is used for the calculation. Consequently, the 2nd order B-spline basis function practically works only as a quadratic function. Therefore, if the image does not include any noise, the estimate value of the fast M-estimation method matches the estimate value of the least-squares method.

The output value when the fast M-estimation method is applied to the GF is explained. The GF can be considered as a weighted least squares method with weights of 1. Therefore, if the surrounding pixels do not include any noises, the output value of the method that combines the fast M-estimation method and the GF is equal to the output value of the GF. On the other hand, if the target pixel is noise and its value is significantly different from the surrounding pixels, the weight distribution for the noise and the weight distribution for the surrounding pixels are divided. If the sum of the weights for the surrounding pixels is larger than the weight for the noise, the output value is calculated from only the surrounding pixels and is not affected by the noise. When the target pixel is an edge, two weight distributions bordering the edge are formed according to the pixel value. In this case, the edge that includes the target pixel is more likely to have a larger sum of weights because of the characteristics of the GF weight function. Therefore, the output value is more likely to be the pixel value on the edge side, including the target pixel, and as a result, the edge is expected to be preserved.

## Proposed method

**Figure 2:**
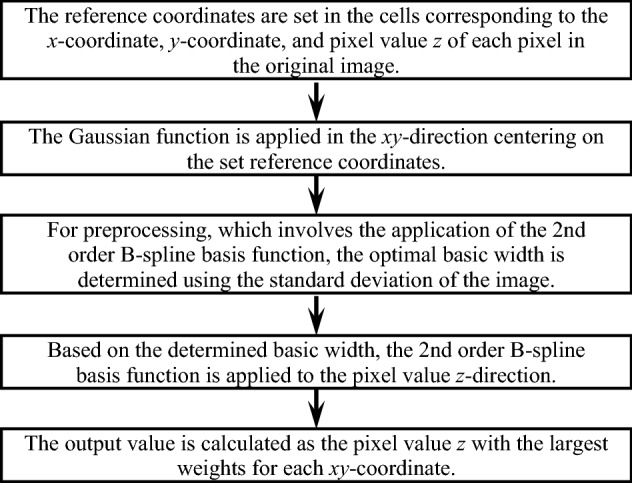
Processing procedure of the abwFMGFI.

**Figure 3:**
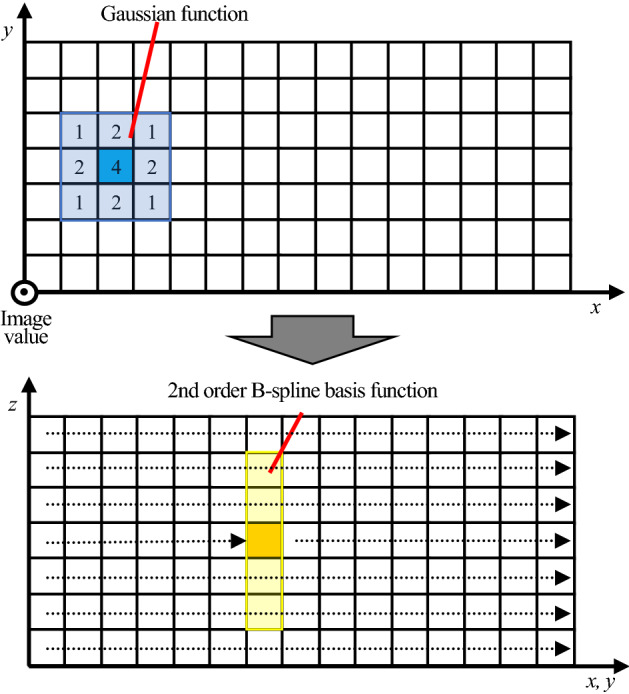
Gaussian function and 2nd order B-spline basis function.

Hereinafter, the proposed method is referred to as the FMGFI with an automatic determination algorithm for basic width (abwFMGFI). The processing procedure for the proposed method is explained in detail. The processing procedure of the abwFMGFI is described by the flowchart in Fig. 2. First, an array is assigned to the pixel value *z* in addition to the *xy*-coordinate. In the case of two-dimensional grayscale images, general image processing is performed using two-dimensional arrays; however, the proposed method uses three-dimensional arrays for this purpose. As depicted in Fig. 3, the Gaussian function and the 2nd order B-spline basis function are applied to three-dimensional arrays. As shown in the first step of Fig. 2, the reference coordinates are set in the cells corresponding to the *x*-coordinate, *y*-coordinate, and pixel value *z* of each pixel in the original image. In the second step of Fig. 2, as shown in the upper part of Fig. 3, the Gaussian function is applied in the *xy*-direction, centering on the set reference coordinates. The general weight function equation of the GF can be expressed as follows.

1 $$f\left(x,y\right)=\frac{1}{2\pi {\sigma }^{2}}exp\left(-\frac{{x}^{2}+{y}^{2}}{2{\sigma }^{2}}\right)$$

The weight matrix equation of the GF (width: 3 × 3) commonly used in image processing can be expressed as follows.

2 $$f\left(x,y\right)=\left[\begin{array}{ccc}\frac{1}{16}& \frac{2}{16}& \frac{1}{16}\\ \frac{2}{16}& \frac{4}{16}& \frac{2}{16}\\ \frac{1}{16}& \frac{2}{16}& \frac{1}{16}\end{array}\right]$$

At that time, in the proposed method, the applied Gaussian function is the value before normalization as follows.

3 $$f\left(x,y\right)=\left[\begin{array}{ccc}1& 2& 1\\ 2& 4& 2\\ 1& 2& 1\end{array}\right]$$

The equation of the general GF convolution operation can be expressed as follows.

4 $$OGF\left(x,y,z\right)= \sum_{x=0}^{width-1}\sum_{y=0}^{height-1}\sum_{a=-i}^{i}\sum_{b=-j}^{j}f(x+a,y+b )IN(x,y)$$

where OGF and IN in the following Eq. (4) are the output of the GF and the coordinates of the original image, respectively.

On the other hand, the convolution operation in the *xy*-direction of the proposed method is as follows because it is applied to the weights.

5 $$AF\left(x,y,z\right)= \sum_{x=0}^{width-1}\sum_{y=0}^{height-1}\sum_{z=0}^{255}\sum_{a=-i}^{i}\sum_{b=-j}^{j}f(x+a,y+b )IN(x,y,z)$$

where AF and IN in the following Eq. (5) are the output of the proposed method and the coordinates of the original image, respectively.

After applying the GF, the 2nd order B-spline basis function is applied to the pixel value *z*-direction, as shown in the lower part of Fig. 3. For calculations on the 2nd order B-spline basis function, the weight array of the pixel value in the *z*-direction is convoluted three times using the box linear filter (hereinafter referred to as the box filter). The size and weight of the box filter are the basic width and 1, respectively.

6 $$ASOB\left(x,y,z\right)= \sum_{x=0}^{width-1}\sum_{y=0}^{height-1}\sum_{z=0}^{255}\sum_{k=-(m-1)/2}^{(m-1)/2}AF\left(x,y,z+k\right)$$

where ASOB and AF in the following Eq. (6) are the output of the 2nd order B-spline basis function and the proposed method, respectively.

The proposed method outputs the z-coordinate with the maximum weight after applying a 2nd order B-spline basis function. At that time, the convolutional integration processing of the 2nd order B-spline basis function is calculated using an integer. In visual evaluation, a larger value of the basic width tends to blur more of the areas that do not contain noise in the image, resulting in lower image quality. When the FMGFI is applied to an image that includes strong noises, the basic width needs to be set to an optimal value to reduce the noises. In previous research, the optimal basic width had to be determined by manual setting of the basic width and likewise human judgment of the filtered results. Therefore, in this research, we develop an algorithm to automatically determine the optimal basic width. In order to solve the above-mentioned problem, we consider that it would be better if the basic width could be automatically increased for denoising when the surrounding image includes noises, and the basic width could be automatically decreased when the surrounding image does not include noises. An algorithm to determine the basic width by the standard deviation of the image is developed to achieve this idea. The standard deviation is calculated from the target pixel and the surrounding pixels in the GF width range. Because the standard deviation is calculated for each pixel, the proposed method can determine the basic width for each pixel. The automatic determination of the basic width corresponds to the third step in Fig. 2. After the basic width is automatically determined in the above flow, as shown by the fourth and final Steps in Fig. 2, the filter processing of the FMGFI is performed. As a result, when noises are included in the surrounding pixels, the proposed method achieves high denoising performance by increasing the basic width. On the other hand, when noise is not included in the surrounding pixels, the proposed method achieves a high reproducibility of the original image by decreasing the basic width.

## Experiments

### Optimal filter size experiment

The proposed method is able to automatically determine the BW, whereas the filter size needs to be set manually. Therefore, the impact of filter size for the performance of the proposed method is studied. To verify the optimal filter size, the proposed method is applied to an image added with salt and pepper noise at several filter sizes in this experiment, the optimal filter size was determined based on visual evaluation and two evaluation parameters. The root mean square error (RMSE) and structural similarity index measure (SSIM) were used. A lower RMSE value and higher SSIM value are considered to indicate a higher reproducibility of the original image. A higher reproducibility of the original image indicates higher denoising and edge preservation performance.

**Figure 4:**
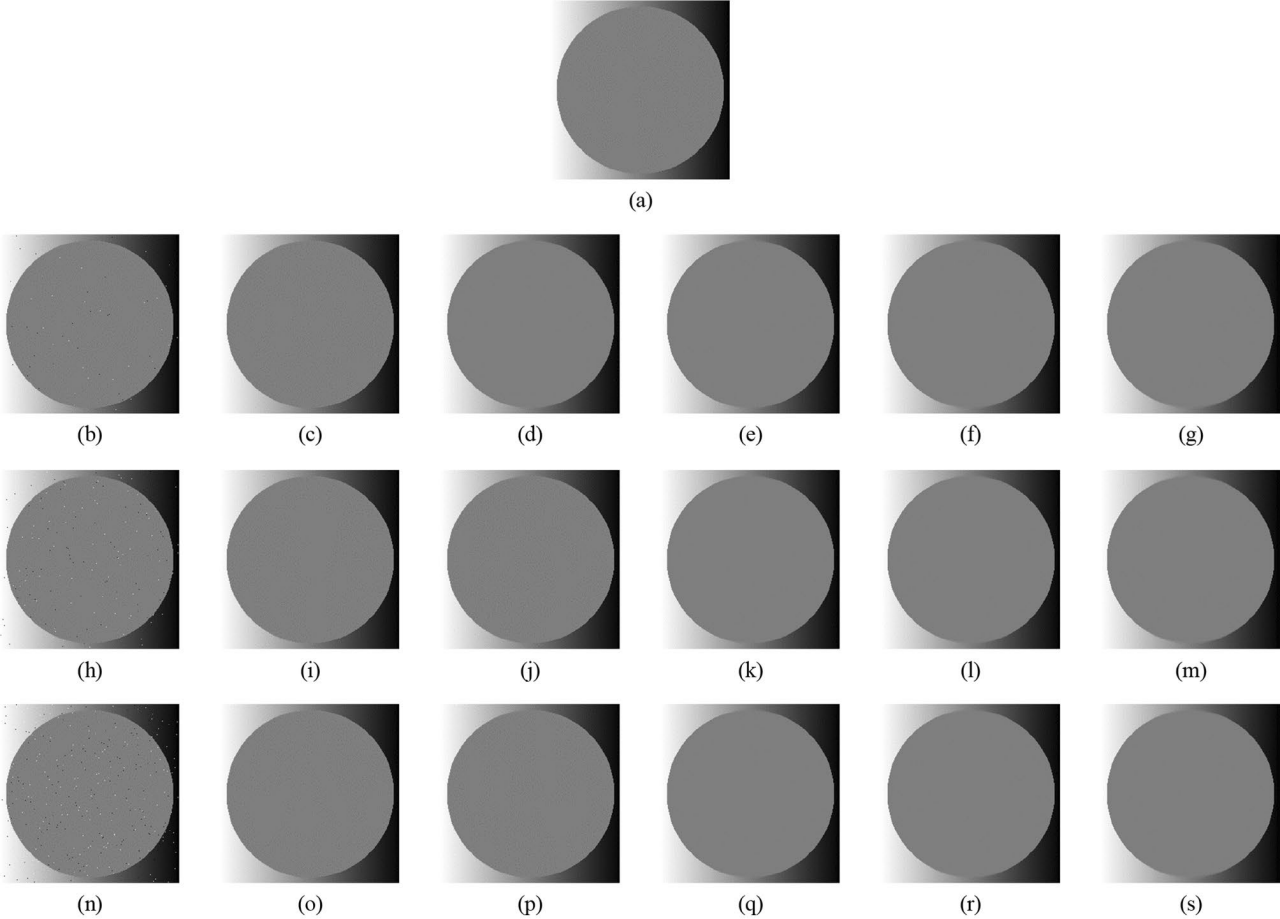
Comparison of noise reduction performance of each filter size and each noise level. (**a**) Original image; (**b**) noise-added image (Noise level = 0.1%); (**c**) filtered image using abwFMGFI (noise level = 0.1%, filter size = 3 × 3); (**d**) filtered image using abwFMGFI (noise level = 0.1%, filter size = 5 × 5); (**e**) filtered image using abwFMGFI (noise level = 0.1%, filter size = 7 × 7); (**f**) filtered image using abwFMGFI (noise level = 0.1%, filter size = 9 × 9); (**g**) filtered image using abwFMGFI (noise level = 0.1%, filter size = 11 × 11); (**h**) noise-added image (noise level = 0.3%); (**i**) filtered image using abwFMGFI (noise level = 0.3%, filter size = 3 × 3); (**j**) filtered image using abwFMGFI (noise level = 0.3%, filter size = 5 × 5); (**k**) filtered image using abwFMGFI (noise level = 0.3%, filter size = 7 × 7); (**l**) filtered image using abwFMGFI (noise level = 0.3%, filter size = 9 × 9); (**m**) filtered image using abwFMGFI (noise level = 0.3%, filter size = 11 × 11); (**n**) noise-added image (noise level = 0.5%); (**o**) filtered image using abwFMGFI (noise level = 0.5%, filter size = 3 × 3); (**p**) filtered image using abwFMGFI (noise level = 0.5%, filter size = 5 × 5); (**q**) filtered image using abwFMGFI (noise level = 0.5%, filter size = 7 × 7); (**r**) filtered image using abwFMGFI (noise level = 0.5%, filter size = 9 × 9); (**s**) filtered image using abwFMGFI (noise level = 0.5%, filter size = 11 × 11).

Figure 4 shows the original image, the noise-added images, and the images after applying each filter to the noise-added image for three patterns of the noise level, respectively. Note that these images are 256 × 256 pixels in size. The filter width in the xy-direction is five patterns (3 × 3, 5 × 5, 7 × 7, 9 × 9, 11 × 11 pixels), and the noise level is three patterns (0.1, 0.3, 0.5%) for the verification. Tables 1 and 2 show the RMSE and SSIM values of each sample image filtered with each filter, respectively. The visual evaluation of Fig. 4 shows that the noises have been removed in all the patterns, whereas the larger filter size blurs the edges. Tables 1 and 2 show that the filter size 3 × 3 has the lowest RMSE value and the highest SSIM value. In addition, owing to the characteristics of the proposed method, a larger GF width increases the computational cost, resulting in a longer processing time. Therefore, based on the results of this experiment, it can be said that the GF width of 3 × 3 pixels is optimal when both image quality and processing time are considered.

**Table 1:** Comparison of the RMSE values of each noise level image filtered using each filter size abwFMGFI.

Noise level (%)	Filter width = 3	Filter width = 5	Filter width = 7	Filter width = 9	Filter width = 11
0.1	0.089	0.178	0.262	0.366	0.603
0.3	0.099	0.165	0.274	0.394	0.647
0.5	0.453	0.702	0.740	0.636	0.823

**Table 2:** Comparison of the SSIM values of each noise level image filtered using each filter size abwFMGFI.

Noise level (%)	Filter width = 3	Filter width = 5	Filter width = 7	Filter width = 9	Filter width = 11
0.1	0.999	0.994	0.992	0.990	0.986
0.3	0.999	0.994	0.992	0.990	0.985
0.5	0.999	0.993	0.991	0.989	0.984

### Filter performance experiment for ground truth signal

**Figure 5:**
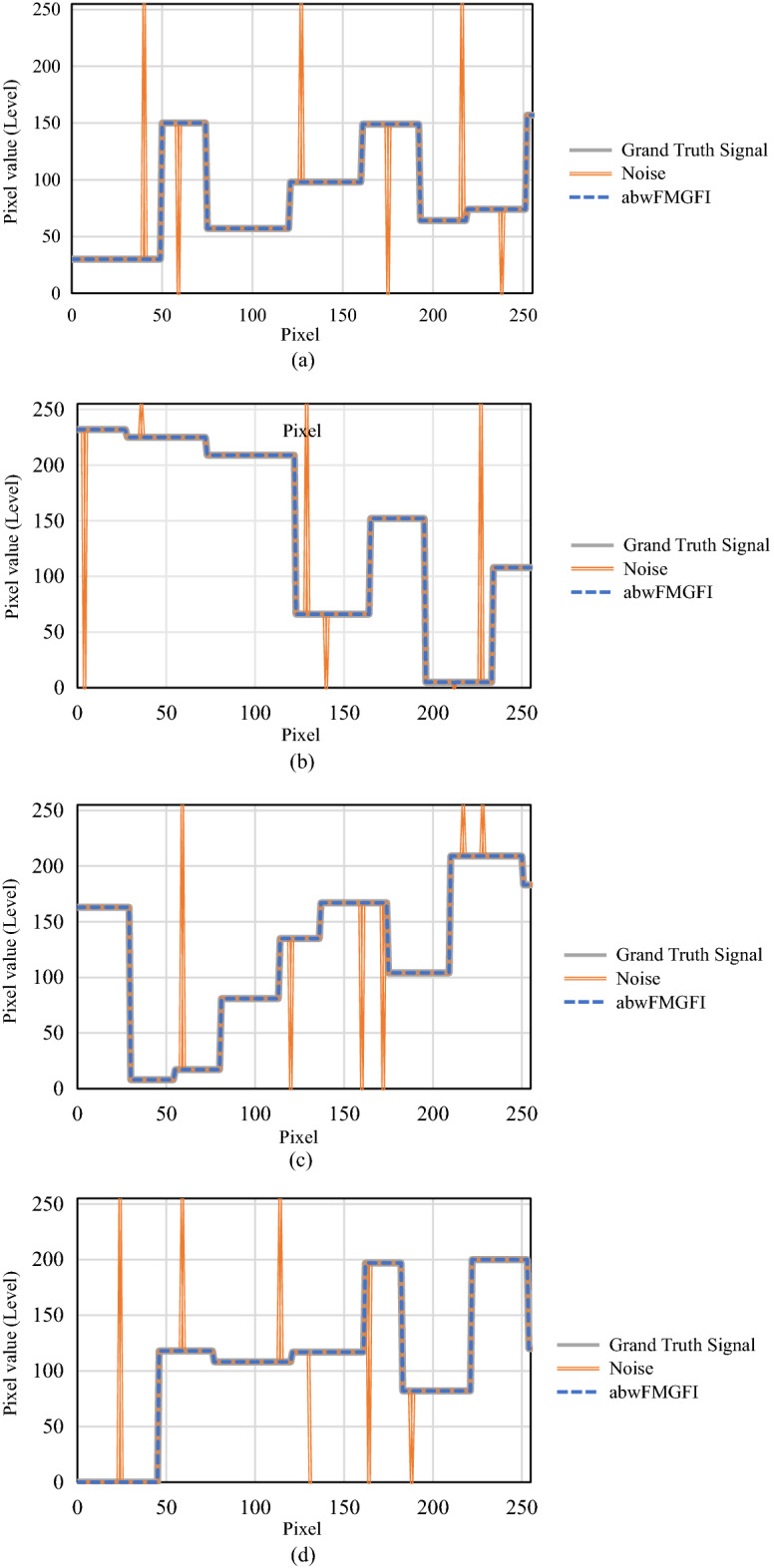
Filtering result for the vp-GTSs using abwFMGFI. (**a**) Pattern 1; (**b**) Pattern 2; (**c**) Pattern 3; (**d**) Pattern 4.

Image-based evaluation cannot provide a clear visual assessment for the robustness of the proposed filter. Therefore, in this experiment, the robustness of the proposed filter is evaluated by adding noise to a ground truth signal with variable patterns (vp-GTS) of the two-dimensional. By presenting the filtered results in the two-dimensional graph, a clear visual evaluation of the proposed filter is achieved. In the experiment, four types of vp-GT signals with the salt-and-pepper noises are validated. Figure 5 shows that the proposed method removes noises and preserves edges in all the patterns. Therefore, we consider that these results demonstrate the robustness of the proposed filter.

### Salt and pepper noise reduction experiment

**Figure 6:**
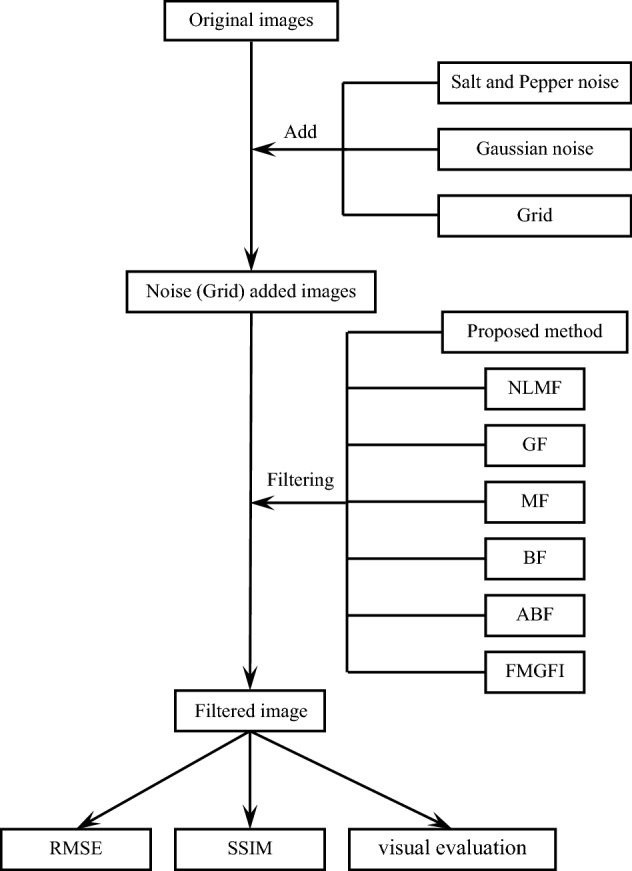
Experimental procedure.

The denoising performance of the proposed method is herein verified. The procedure of the experiments illustrated using a flowchart in Fig. 6, which also includes the experimental procedures that will be discussed later, in “Gaussian noise reduction experiment” and “Edge preservation experiment”. In this experiment, the denoising performance of each filter on the salt-and-pepper noise-added sample images was compared based on visual evaluation, RMSE and SSIM. The filters used in the experiments are the proposed method, NLMF, GF, MF, BF, ABF, and FMGFI. The sample images used are one geometric image, and 8 types of images from the USC-SIPI Image database and SIDBA (hereinafter referred to as standard images). The sample images are each of 256 × 256 pixels and each filter size is of 3 × 3 pixels. To avoid implementation errors, the NLMF, GF, MF, BF, and ABF are implemented using OpenCV 4.3.2 and 2.4.13. The parameter values are the same as those used in reference^40^ and as recommended by OpenCV. The basic widths of the FMGFI are 1, 3, 5, 11, 21, and 51 levels.

**Figure 7:**
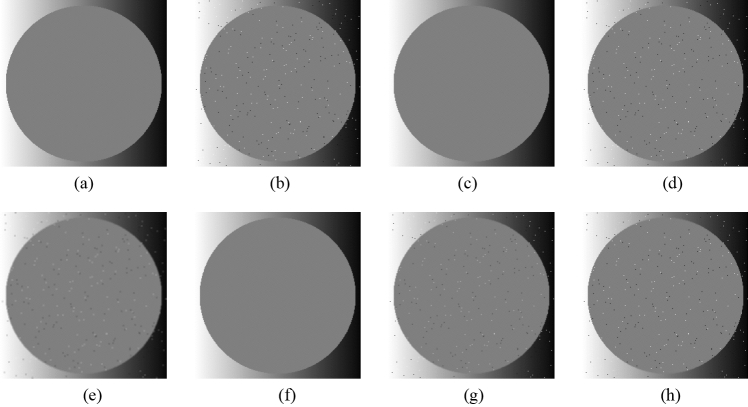
Comparison of the denoising performance of each filter against the salt-and-pepper noise (“Geometric pattern”). (**a**) Original image; (**b**) noise-added image; (**c**) filtered image using abwFMGFI; (**d**) filtered image using NLMF (templateWindowSize: 21, searchWindowSize: 7); (**e**) filtered image using GF (σ_x: 5, σ_y: 5); (**f**) filtered image using MF; (**g**) filtered image using BF (σ_c: 35, σ_s: 5); (**h**) filtered image using ABF (σ_s: 7, max_σ_c: 35); where σ_x, σ_y, σ_c, σ_s, and max_σ_c represent sigmaX, sigmaY, sigmaColor, sigmaSpace, and maxSigmaColor in OpenCV, respectively.

**Figure 8:**
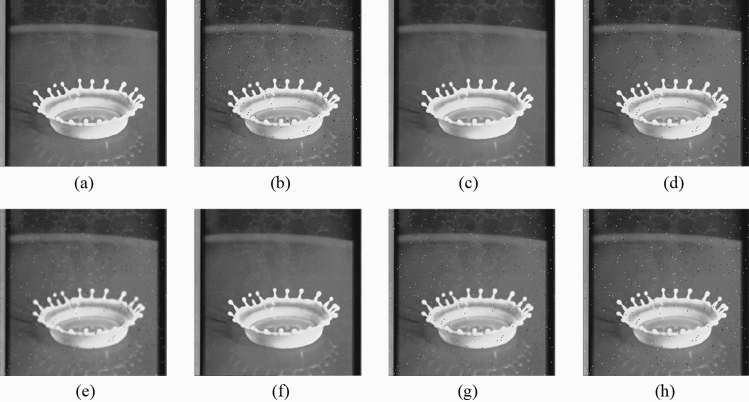
Comparison of denoising performance of each filter against the salt-and-pepper noise (“Milkdrop”). (**a**) Original image; (**b**) noise-added image; (**c**) filtered image using abwFMGFI; (**d**) filtered image using NLMF (templateWindowSize: 21, searchWindowSize: 7); (**e**) filtered image using GF (σ_x: 5, σ_y: 5); (**f**) filtered image using MF; (**g**) filtered image using BF (σ_c: 35, σ_s: 5); (**h**) filtered image using ABF (σ_s: 7, max_σ_c: 35).

**Figure 9:**
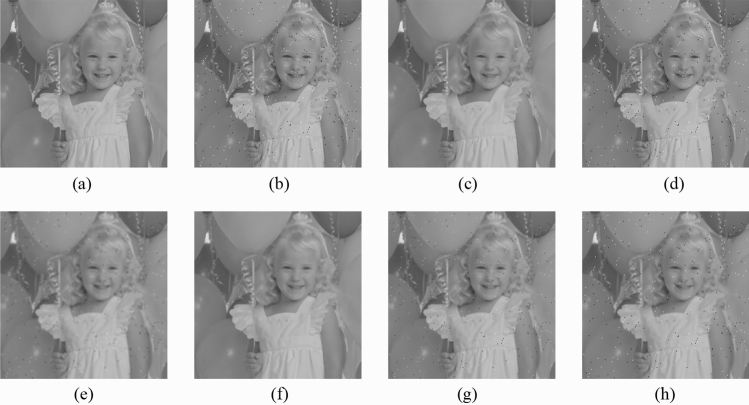
Comparison of denoising performance of each filter against the salt-and-pepper noise (“Balloon”). (**a**) Original image; (**b**) noise-added image; (**c**) filtered image using abwFMGFI; (**d**) filtered image using NLMF (templateWindowSize: 21, searchWindowSize: 7); (**e**) filtered image using GF (σ_x: 5, σ_y: 5); (**f**) filtered image using MF; (**g**) filtered image using BF (σ_c: 35, σ_s: 5); (**h**) filtered image using ABF (σ_s: 7, max_σ_c: 35).

**Figure 10:**
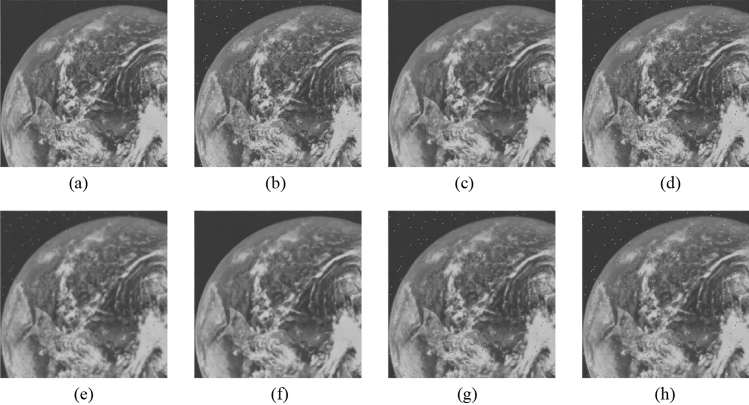
Comparison of denoising performance of each filter against the salt-and-pepper noise (“Earth”). (**a**) Original image; (**b**) noise-added image; (**c**) filtered image using abwFMGFI; (**d**) filtered image using NLMF (templateWindowSize: 21, searchWindowSize: 7); (**e**) filtered image using GF (σ_x: 5, σ_y: 5); (**f**) Filtered image using MF; (**g**) filtered image using BF (σ_c: 35, σ_s: 5); (**h**) filtered image using ABF (σ_s: 7, max_σ_c: 35).

**Figure 11:**
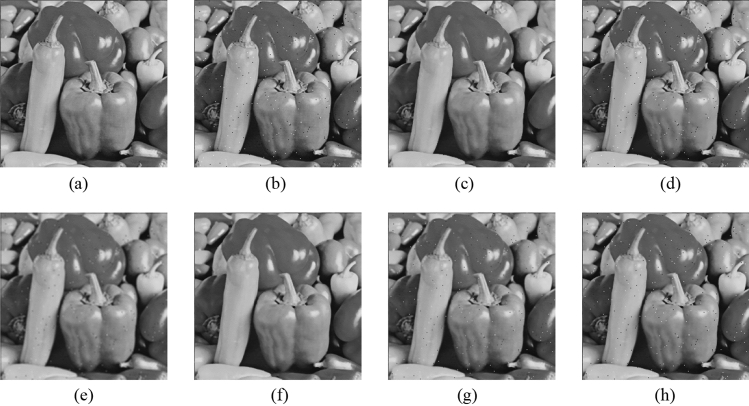
Comparison of denoising performance of each filter against the salt-and-pepper noise (“Pepper”). (**a**) Original image; (**b**) noise-added image; (**c**) filtered image using abwFMGFI; (**d**) filtered image using NLMF (templateWindowSize: 21, searchWindowSize: 7); (**e**) filtered image using GF (σ_x: 5, σ_y: 5); (**f**) filtered image using MF; (**g**) filtered image using BF (σ_c: 35, σ_s: 5); (**h**) filtered image using ABF (σ_s: 7, max_σ_c: 35).

First, the proposed method is compared with the widely used filters, such as the NLMF, GF, MF, BF, and ABF. Figures 7, 8, 9, 10 and 11 show the original image, noise-added image, and image after applying each filter to the noise-added image for 12 types of sample images. Tables 3 and 4 show the RMSE and SSIM values, respectively, of each sample image filtered using each filter. In Tables 3 and 4, the values for the filtered images with remaining noise or blurred edges are shown in gray. Visual evaluations confirmed that only the proposed method achieves simultaneous denoising and edge preservation. The MF is able to remove noise, however, blurs the edges. The GF reduced the noise, however, does not remove the noise and strongly blurs the entire image. The filtered results using the NLMF, BF, and ABF showed little change and could not reduce the noise. As presented in Table 3, the RMSE value of the proposed method is the lowest for all the sample images except the "milkdrop" image. The MF-applied "milkdrop" is blurred over the entire image, and the deviation of the RMSE from the proposed method is a small deviation of − 1.7%. As presented in Table 4, the RMSE value of the proposed method is the lowest for all the sample images except for the "Aerial" image. The noise remained in the NLMF-applied " Aerial", and the deviation of the SSIM from the proposed method is a small deviation of 2.0%. These results show that the MF and the NLMF cannot be said to have a high denoising performance based solely on the RMSE values. Further, we consider that the proposed method has a higher denoising performance against the salt-and-pepper noise as compared to the NLMF, GF, MF, BF, and ABF.

**Table 3:** Comparison of the RMSE values of each sample image filtered using each filter (against the salt-and-pepper noise).

Image name	abwFMGFI	NLMF	GF	MF	BF	ABF
Geometric pattern	0.453	10.007	5.433	0.779	7.394	9.934
Milkdrop	2.841	10.455	6.577	2.794	8.326	10.452
Balloon	2.183	9.312	4.862	3.058	5.904	9.459
Earth	3.460	10.056	6.803	5.116	7.859	10.705
Pepper	4.052	9.844	8.664	5.234	8.106	10.199

**Table 4:** Comparison of the SSIM values of each sample image filtered using each filter (against the salt-and-pepper noise).

Image name	abwFMGFI	NLMF	GF	MF	BF	ABF
Geometric pattern	0.999	0.627	0.561	0.999	0.582	0.622
Milkdrop	0.875	0.608	0.655	0.785	0.740	0.645
Balloon	0.891	0.621	0.679	0.831	0.726	0.639
Earth	0.926	0.787	0.772	0.862	0.828	0.748
Pepper	0.930	0.768	0.763	0.876	0.820	0.765

**Figure 12:**
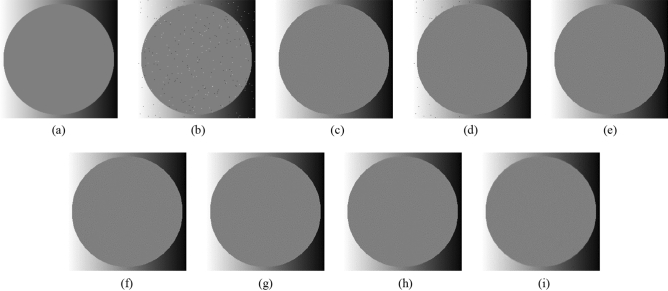
Comparison of denoising performance of each filter against the salt-and-pepper noise (“Geometric pattern”). (**a**) Original image; (**b**) noise-added image; (**c**) filtered image using abwFMGFI; (**d**) filtered image using FMGFI (BW = 1 level); (**e**) filtered image using FMGFI (BW = 3 level); (**f**) filtered image using FMGFI (BW = 5 level); (**g**) filtered image using FMGFI (BW = 11 level); (**h**) filtered image using FMGFI (BW = 21 level); (**i**) filtered image using FMGFI (BW = 51 level).

**Figure 13:**
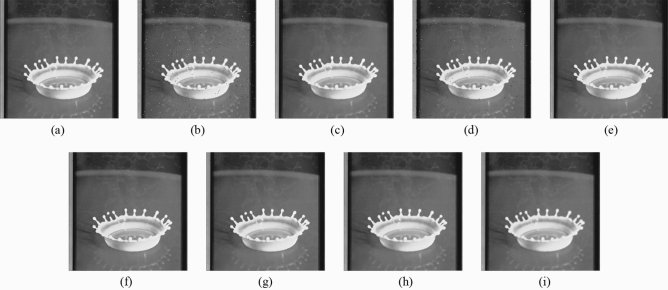
Comparison of denoising performance of each filter against the salt-and-pepper noise (“Milkdrop”). (**a**) Original image; (**b**) noise-added image; (**c**) filtered image using abwFMGFI; (**d**) filtered image using FMGFI (BW = 1 level); (**e**) filtered image using FMGFI (BW = 3 level); (**f**) filtered image using FMGFI (BW = 5 level); (**g**) filtered image using FMGFI (BW = 11 level); (**h**) filtered image using FMGFI (BW = 21 level); (**i**) filtered image using FMGFI (BW = 51 level).

**Figure 14:**
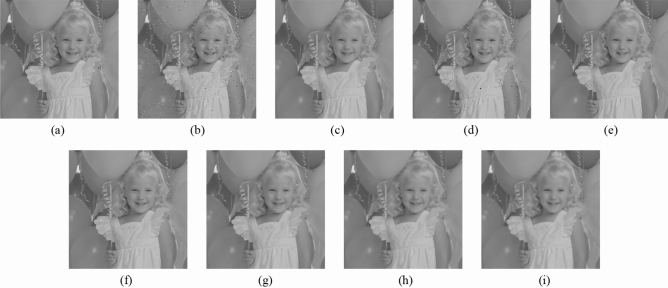
Comparison of denoising performance of each filter against the salt-and-pepper noise (“Balloon”). (**a**) Original image; (**b**) noise-added image; (**c**) filtered image using abwFMGFI; (**d**) filtered image using FMGFI (BW = 1 level); (**e**) filtered image using FMGFI (BW = 3 level); (**f**) filtered image using FMGFI (BW = 5 level); (**g**) filtered image using FMGFI (BW = 11 level); (**h**) filtered image using FMGFI (BW = 21 level); (**i**) filtered image using FMGFI (BW = 51 level).

**Figure 15:**
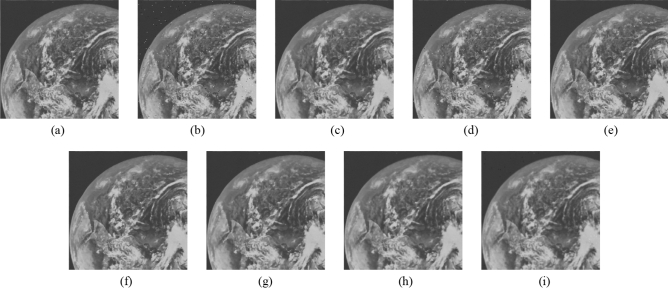
Comparison of denoising performance of each filter against the salt-and-pepper noise (“Earth”). (**a**) Original image; (**b**) noise-added image; (**c**) filtered image using abwFMGFI; (**d**) filtered image using FMGFI (BW = 1 level); (**e**) filtered image using FMGFI (BW = 3 level); (**f**) filtered image using FMGFI (BW = 5 level); (**g**) filtered image using FMGFI (BW = 11 level); (**h**) filtered image using FMGFI (BW = 21 level); (**i**) filtered image using FMGFI (BW = 51 level).

**Figure 16:**
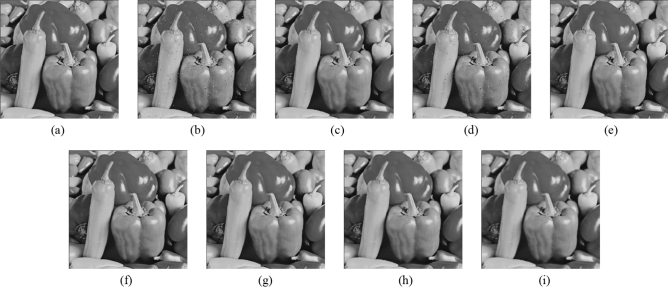
Comparison of denoising performance of each filter against the salt-and-pepper noise (“Pepper”). (**a**) Original image; (**b**) noise-added image; (**c**) filtered image using abwFMGFI; (**d**) filtered image using FMGFI (BW = 1 level); (**e**) filtered image using FMGFI (BW = 3 level); (**f**) filtered image using FMGFI (BW = 5 level); (**g**) filtered image using FMGFI (BW = 11 level); (**h**) filtered image using FMGFI (BW = 21 level); (**i**) filtered image using FMGFI (BW = 51 level).

Subsequently, the proposed method and the FMGFI are compared. Figures 12, 13, 14, 15 and 16 show the original image, the noise-added image, and the image after applying each filter to the noise-added image for 12 types of sample image. Tables 5 and 6 show the RMSE and SSIM values, respectively, of each sample image filtered using each filter. In Tables 5 and 6, the numerical values are indicated in gray if the filtered image has residual noise or blurred edges. The proposed method and the FMGFI (basic width = 11, 21 level) are confirmed to have achieved simultaneous denoising and edge preservation in visual evaluation. The filtered results of the FMGFI (basic width = 1, 3, 5 level) showed some remaining noise, and the FMGFI (basic width = 51 level) blurred the edges. As presented in Table 5, the proposed method shows the lowest RMSE value among the filters with confirmed denoising properties. As presented in Table 6, the proposed method shows the highest SSIM value among the filters with confirmed denoising properties. Therefore, we consider that the proposed method has a higher denoising performance against the salt-and-pepper noise than the FMGFI of any basic width.

**Table 5:** Comparison of the RMSE values of each sample image filtered using the variable level of BW of FMGFI (against the salt-and-pepper noise).

Image name	abwFMGFI	FMGFI					
		BW = 1 level	BW = 3 level	BW = 5 level	BW = 11 level	BW = 21 level	BW = 51 level
Geometric pattern	0.453	5.088	0.646	0.647	0.490	0.644	2.014
Milkdrop	2.841	8.127	3.483	3.521	3.769	3.423	3.697
Balloon	2.183	6.664	2.901	2.582	2.476	2.579	3.022
Earth	3.460	8.514	5.535	4.552	3.776	4.004	4.558
Pepper	4.052	8.334	4.713	4.428	4.768	4.528	5.032

**Table 6:** Comparison of the SSIM values of each sample image filtered using the variable level of BW of FMGFI (against the salt-and-pepper noise).

Image name	abwFMGFI	FMGFI					
		BW = 1 level	BW = 3 level	BW = 5 level	BW = 11 level	BW = 21 level	BW = 51 level
Geometric pattern	0.999	0.953	0.999	0.999	0.997	0.993	0.695
Milkdrop	0.930	0.871	0.926	0.922	0.915	0.913	0.894
Balloon	0.891	0.844	0.887	0.885	0.879	0.871	0.841
Earth	0.926	0.878	0.911	0.916	0.915	0.905	0.887
Pepper	0.892	0.843	0.888	0.889	0.881	0.873	0.845

### Gaussian noise reduction experiment

The noise reduction experiment is performed against Gaussian noise. In the experiment, the denoising performance of each filter on the-Gaussian noise-added (standard deviation 10) sample images is compared based on visual evaluation and using two evaluation parameters. The filters, filter parameters, sample images, and evaluation parameters used in this experiment are the same as those use in “Salt and pepper noise reduction experiment”.

**Figure 17:**
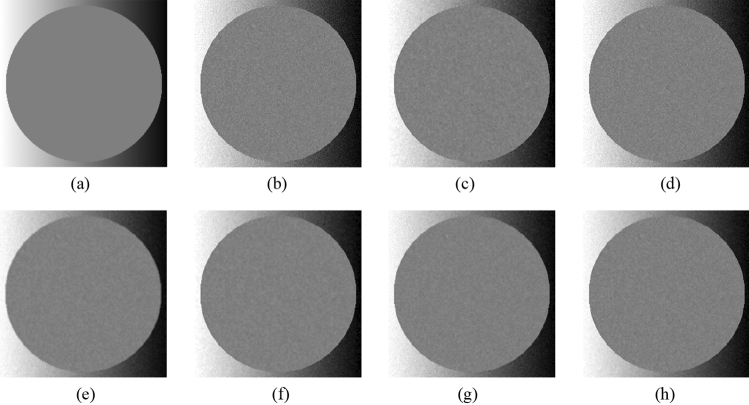
Comparison of denoising performance of each filter against the Gaussian noise (“Geometric pattern”). (**a**) Original image; (**b**) noise-added image; (**c**) filtered image using abwFMGFI; (**d**) filtered image using NLMF (templateWindowSize: 21, searchWindowSize: 7); (**e**) filtered image using GF (σ_x: 5, σ_y: 5); (**f**) filtered image using MF; (**g**) filtered image using BF (σ_c: 35, σ_s: 5); (**h**) filtered image using ABF (σ_s: 7, max_σ_c: 35).

**Figure 18:**
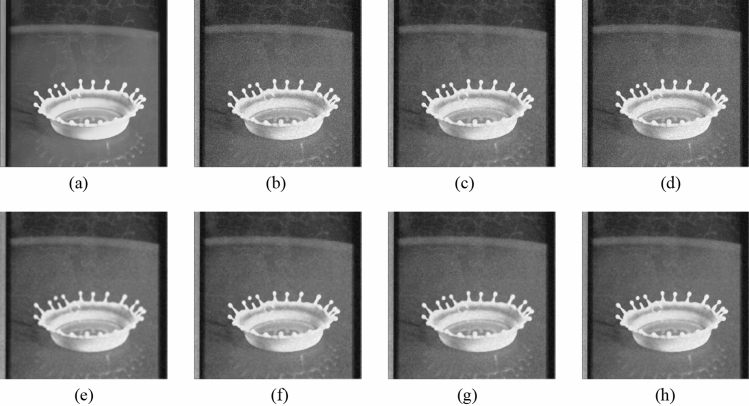
Comparison of denoising performance of each filter against the Gaussian noise (“Milkdrop”). (**a**) Original image; (**b**) noise-added image; (**c**) filtered image using abwFMGFI; (**d**) filtered image using NLMF (templateWindowSize: 21, searchWindowSize: 7); (**e**) filtered image using GF (σ_x: 5, σ_y: 5); (**f**) filtered image using MF; (**g**) filtered image using BF (σ_c: 35, σ_s: 5); (**h**) filtered image using ABF (σ_s: 7, max_σ_c: 35).

**Figure 19:**
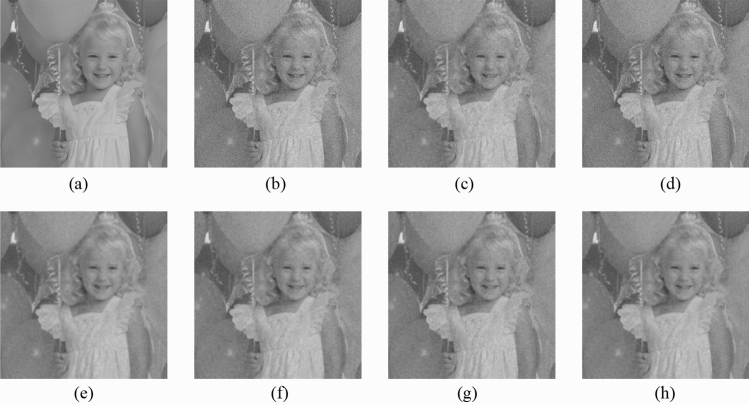
Comparison of denoising performance of each filter against the Gaussian noise (“Balloon”). (**a**) Original image; (**b**) noise-added image; (**c**) filtered image using abwFMGFI; (**d**) filtered image using NLMF (templateWindowSize: 21, searchWindowSize: 7); (**e**) filtered image using GF (σ_x: 5, σ_y: 5); (**f**) filtered image using MF; (**g**) filtered image using BF (σ_c: 35, σ_s: 5); (**h**) filtered image using ABF (σ_s: 7, max_σ_c: 35).

**Figure 20:**
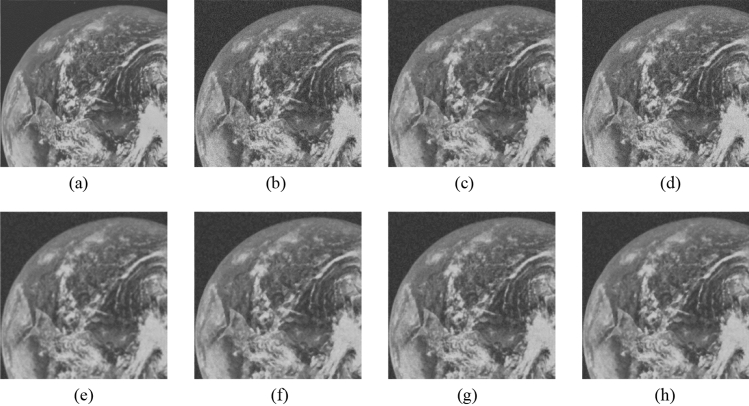
Comparison of denoising performance of each filter against the Gaussian noise (“Earth”). (**a**) Original image; (**b**) noise-added image; (**c**) filtered image using abwFMGFI; (**d**) filtered image using NLMF (templateWindowSize: 21, searchWindowSize: 7); (**e**) filtered image using GF (σ_x: 5, σ_y: 5); (**f**) filtered image using MF; (**g**) filtered image using BF (σ_c: 35, σ_s: 5); (**h**) filtered image using ABF (σ_s: 7, max_σ_c: 35).

**Figure 21:**
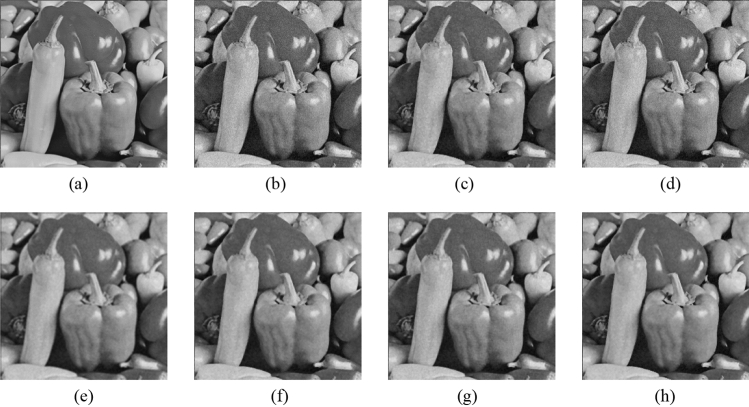
Comparison of denoising performance of each filter against the Gaussian noise (“Pepper”). (**a**) Original image; (**b**) noise-added image; (**c**) filtered image using abwFMGFI; (**d**) filtered image using NLMF (templateWindowSize: 21, searchWindowSize: 7); (**e**) filtered image using GF (σ_x: 5, σ_y: 5); (**f**) filtered image using MF; (**g**) filtered image using BF (σ_c: 35, σ_s: 5); (**h**) filtered image using ABF (σ_s: 7, max_σ_c: 35).

First, as before, the proposed method is compared with the NLMF, GF, MF, BF, and ABF. Figures 17, 18, 19, 20 and 21 show the original image, noise-added image, and image of applying each filter to the noise-added image for 12 types of sample images. Tables 7 and 8 show the RMSE and SSIM values, respectively, of each sample image filtered using each filter. In Tables 7 and 8, the values for the filtered images with blurred edges are indicated in gray. Visual evaluation confirmed that all the filters reduced the noise; however, all the images are rough-textured owing to the remine of noise, which could not be entirely removed. In addition, the filtered results obtained by the proposed method and the NLMF and ABF are little different by visual evaluation. The results also showed that the GF, BF, and MF blur the edges and decreased the image quality. As presented in Tables 7 and 8, the MF, BF, and ABF tend to have lower RMSE values and higher SSIM values than those of the proposed method. However, it cannot be considered that the MF and BF are highly reproducible in the original image because the MF and BF blur the edges. In addition, the NLMF tended to have higher RMSE values and lower SSIM values than those of the proposed method. From the above, we consider that the denoising performance of the proposed method against Gaussian noise is inferior to that of the ABF in the RMSE evaluation, although it is the same as that of the NLMF and ABF as per the visual evaluation results.

**Table 7:** Comparison of the RMSE values of each sample image filtered using each filter (against the Gaussian noise).

Image name	abwFMGFI	NLMF	GF	MF	BF	ABF
Geometric pattern	5.735	9.857	5.458	4.238	4.999	3.826
Milkdrop	6.703	9.933	6.460	5.382	5.398	4.809
Balloon	6.552	10.009	5.017	5.369	5.184	4.529
Earth	7.675	10.019	6.743	7.023	5.897	5.862
Pepper	7.557	9.987	8.675	7.097	6.259	5.619

**Table 8:** Comparison of the SSIM values of each sample image filtered using each filter (against the Gaussian noise).

Image name	abwFMGFI	NLMF	GF	MF	BF	ABF
Geometric pattern	0.127	0.085	0.162	0.155	0.138	0.160
Milkdrop	0.497	0.407	0.561	0.543	0.565	0.597
Balloon	0.415	0.315	0.511	0.471	0.480	0.528
Earth	0.697	0.627	0.729	0.717	0.755	0.759
Pepper	0.653	0.559	0.700	0.696	0.711	0.746

**Figure 22:**
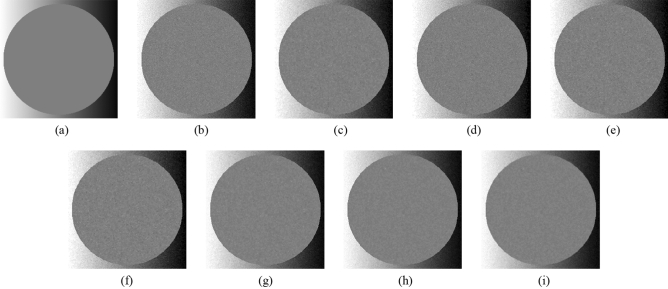
Comparison of denoising performance of each filter against the Gaussian noise (“Geometric pattern”). (**a**) Original image; (**b**) noise-added image; (**c**) filtered image using abwFMGFI; (**d**) filtered image using FMGFI (BW = 1 level); (**e**) filtered image using FMGFI (BW = 3 level); (**f**) filtered image using FMGFI (BW = 5 level); (**g**) filtered image using FMGFI (BW = 11 level); (**h**) filtered image using FMGFI (BW = 21 level); (**i**) filtered image using FMGFI (BW = 51 level).

**Figure 23:**
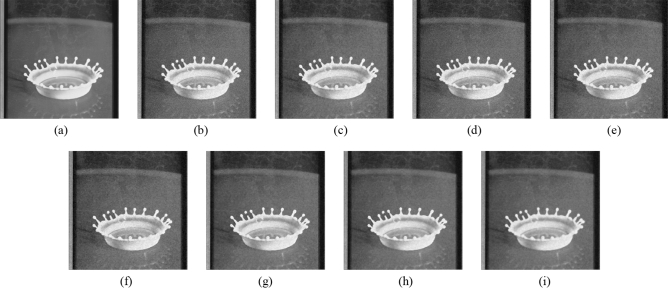
Comparison of denoising performance of each filter against the Gaussian noise (“Milkdrop”). (**a**) Original image; (**b**) noise-added image; (**c**) filtered image using abwFMGFI; (**d**) filtered image using FMGFI (BW = 1 level); (**e**) filtered image using FMGFI (BW = 3 level); (**f**) filtered image using FMGFI (BW = 5 level); (**g**) filtered image using FMGFI (BW = 11 level); (**h**) filtered image using FMGFI (BW = 21 level); (**i**) filtered image using FMGFI (BW = 51 level).

**Figure 24:**
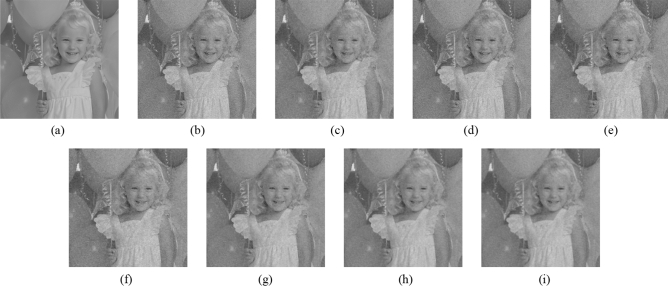
Comparison of denoising performance of each filter against the Gaussian noise (“Balloon”). (**a**) Original image; (**b**) noise-added image; (**c**) filtered image using abwFMGFI; (**d**) filtered image using FMGFI (BW = 1 level); (**e**) filtered image using FMGFI (BW = 3 level); (**f**) filtered image using FMGFI (BW = 5 level); (**g**) filtered image using FMGFI (BW = 11 level); (**h**) filtered image using FMGFI (BW = 21 level); (**i**) filtered image using FMGFI (BW = 51 level).

**Figure 25:**
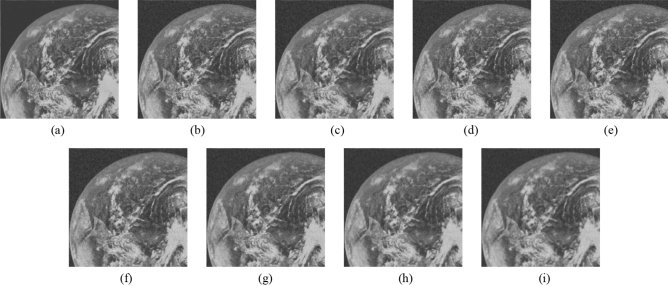
Comparison of denoising performance of each filter against the Gaussian noise (“Earth”). (**a**) Original image; (**b**) noise-added image; (**c**) Filtered image using abwFMGFI; (**d**) filtered image using FMGFI (BW = 1 level); (**e**) filtered image using FMGFI (BW = 3 level); (**f**) filtered image using FMGFI (BW = 5 level); (**g**) filtered image using FMGFI (BW = 11 level); (**h**) filtered image using FMGFI (BW = 21 level); (**i**) filtered image using FMGFI (BW = 51 level).

**Figure 26:**
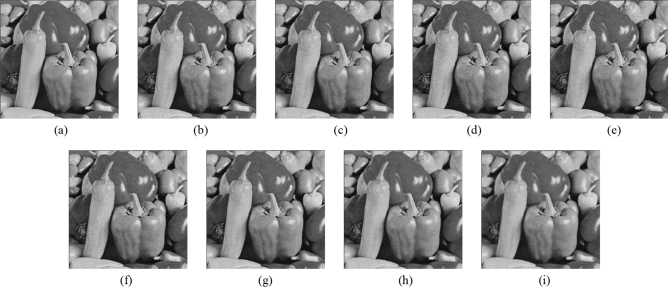
Comparison of denoising performance of each filter against the Gaussian noise (“Pepper”). (**a**) Original image; (**b**) noise-added image; (**c**) filtered image using abwFMGFI; (**d**) filtered image using FMGFI (BW = 1 level); (**e**) filtered image using FMGFI (BW = 3 level); (**f**) filtered image using FMGFI (BW = 5 level); (**g**) filtered image using FMGFI (BW = 11 level); (**h**) filtered image using FMGFI (BW = 21 level); (**i**) filtered image using FMGFI (BW = 51 level).

Subsequently, the proposed method and the FMGFI are compared. Figures 22, 23, 24, 25 and 26 show the original image, noise-added image, and image of applying each filter to the noise-added image for 12 types of sample image. Tables 9 and 10 shows the RMSE and SSIM values, respectively, of each sample image filtered using each filter. In Tables 9 and 10, the values for the filtered images with little change or blurred edges are shown in gray. In the visual evaluation, the proposed method and FMGFI (basic width = 3, 5, 11, 21 level) reduced the noise to a comparable level, whereas the rough texture remains because the noise is not entirely removed. The FMGFI (basic width = 51 level) blurs the edges, and the FMGFI (basic width = 1 level) shows little change and could not reduce the noise. As presented in Tables 9 and 10, the FMGFI (basic width = 11, 21, 51 level) tends to have lower RMSE values and higher SSIM values than those of the proposed method. However, it cannot be considered that the FMGFI (basic width = 51 level) is high in the reproduction of the original image because the FMGFI (basic width = 51 level) blurs the edges. From the above, we consider that the denoising performance of the proposed method against the Gaussian noise is the same as that of the FMGFI in visual evaluation.

**Table 9:** Comparison of the RMSE values of each sample image filtered using the variable level of BW of FMGFI (against the Gaussian noise).

Image name	abwFMGFI	FMGFI					
		BW = 1 level	BW = 3 level	BW = 5 level	BW = 11 level	BW = 21 level	BW = 51 level
Geometric pattern	5.735	9.599	7.764	6.796	4.542	3.851	4.237
Milkdrop	6.703	9.756	8.341	7.601	5.966	5.121	5.162
Balloon	6.552	9.839	8.338	7.507	5.572	4.668	4.754
Earth	7.675	9.992	9.184	8.743	7.204	5.966	5.869
Pepper	7.557	9.983	8.898	8.441	7.268	6.259	6.243

**Table 10:** Comparison of the SSIM values of each sample image filtered using the variable level of BW of FMGFI (against the Gaussian noise).

Image name	abwFMGFI	FMGFI					
		BW = 1 level	BW = 3 level	BW = 5 level	BW = 11 level	BW = 21 level	BW = 51 level
Geometric pattern	0.127	0.087	0.103	0.114	0.151	0.164	0.160
Milkdrop	0.497	0.410	0.439	0.463	0.559	0.607	0.603
Balloon	0.415	0.319	0.351	0.379	0.473	0.519	0.518
Earth	0.697	0.628	0.647	0.664	0.730	0.767	0.767
Pepper	0.653	0.561	0.594	0.620	0.702	0.743	0.741

### Edge preservation experiment

The edge preservation performance of the proposed method is verified. In this experiment, the edge preservation performances of each filter on the wafer-added sample image is compared based on visual evaluation and using two evaluation parameters. Because edge preservation is easy to achieve in the case of a geometric image, the images filtered using each filter differed very slightly. Therefore, for the sample images in this experiment, the edge preservation performance is verified not only by the geometric image but also by the images added the grid to the standard images used in “Salt and pepper noise reduction experiment”. The RMSE and the SSIM are calculated using the differences between the images with the grid and those after each filter was applied. The filters, filter parameters, sample images, and evaluation parameters used in this experiment are the same as those use in “Salt and pepper noise reduction experiment”.

**Figure 27:**
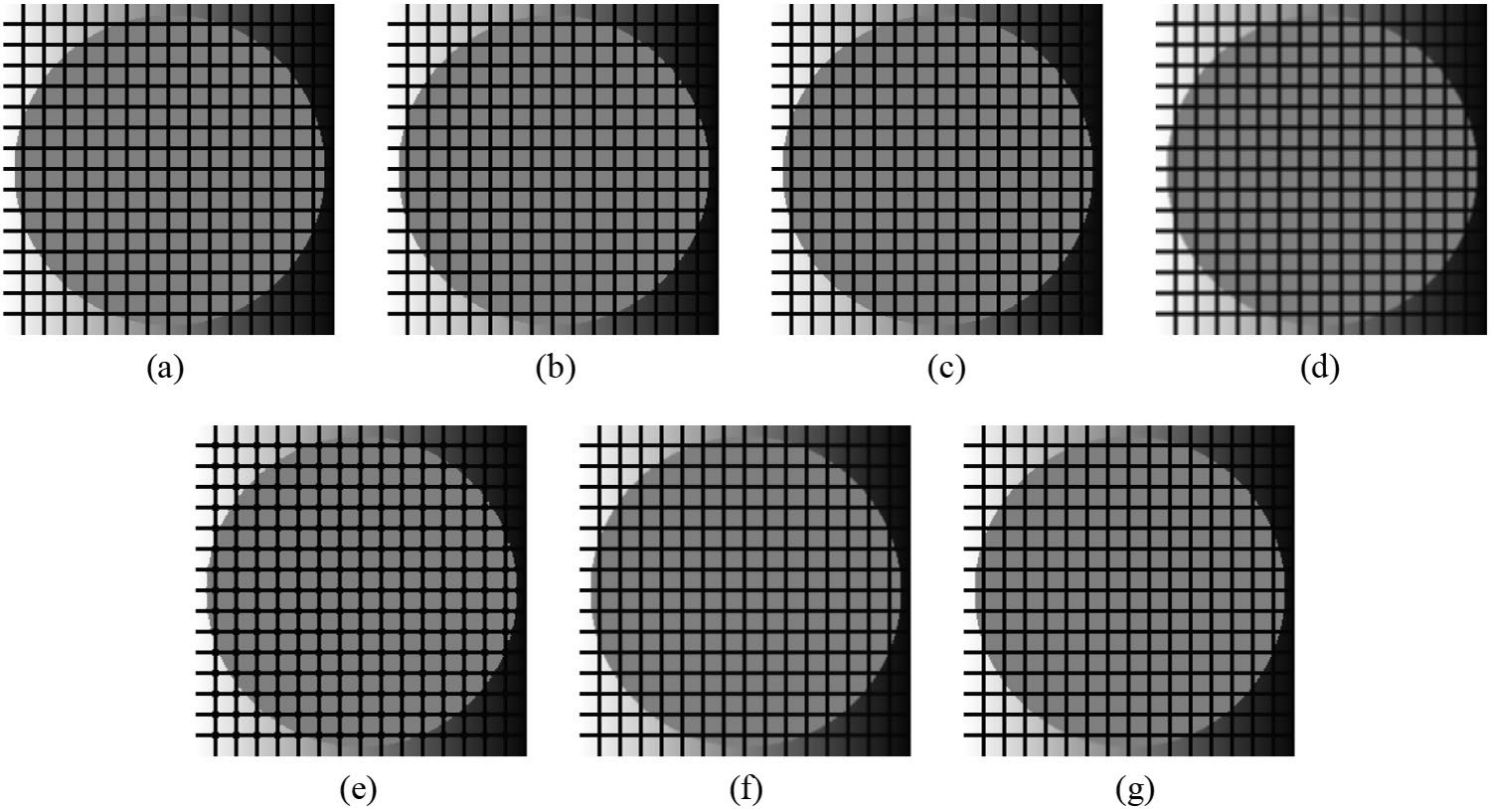
Comparison of edge preservation performance of each filter (“Geometric pattern”). (**a**) Grid-added image; (**b**) filtered image using abwFMGFI; (**c**) filtered image using NLMF (templateWindowSize: 21, searchWindowSize: 7); (**d**) filtered image using GF (σ_x: 5, σ_y: 5); (**e**) filtered image using MF; (**f**) filtered image using BF (σ_c: 35, σ_s: 5); (**g**) filtered image using ABF (σ_s: 7, max_σ_c: 35).

**Figure 28:**
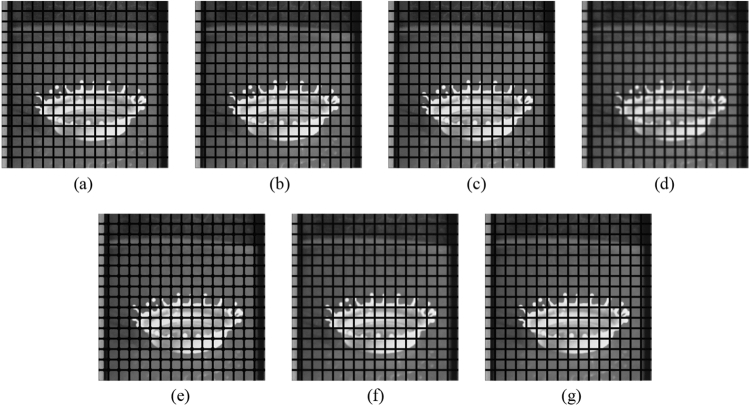
Comparison of edge preservation performance of each filter (“Milkdrop”). (**a**) Grid-added image; (**b**) filtered image using abwFMGFI; (**c**) filtered image using NLMF (templateWindowSize: 21, searchWindowSize: 7); (**d**) filtered image using GF (σ_x: 5, σ_y: 5); (**e**) filtered image using MF; (**f**) filtered image using BF (σ_c: 35, σ_s: 5); (**g**) filtered image using ABF (σ_s: 7, max_σ_c: 35).

**Figure 29:**
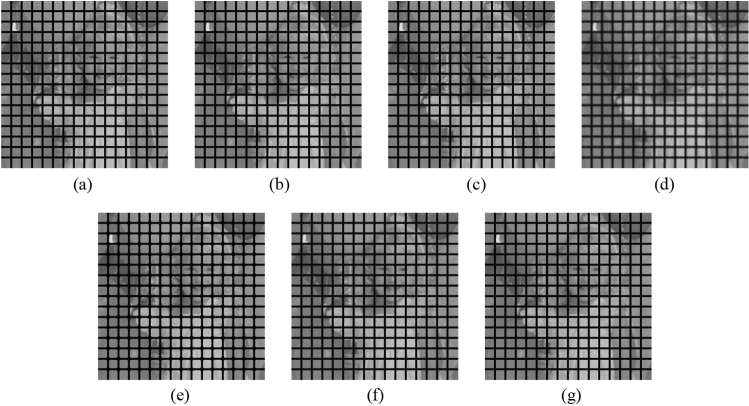
Comparison of edge preservation performance of each filter (“Balloon”). (**a**) Grid-added image; (**b**) filtered image using abwFMGFI; (**c**) filtered image using NLMF (templateWindowSize: 21, searchWindowSize: 7); (**d**) filtered image using GF (σ_x: 5, σ_y: 5); (**e**) filtered image using MF; (**f**) filtered image using BF (σ_c: 35, σ_s: 5); (**g**) filtered image using ABF (σ_s: 7, max_σ_c: 35).

**Figure 30:**
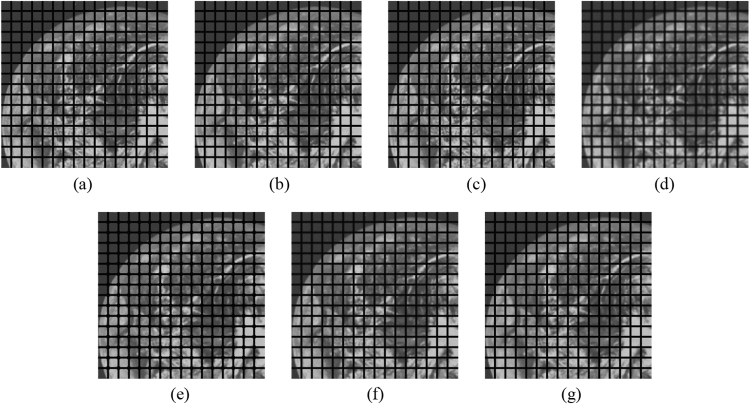
Comparison of edge preservation performance of each filter (“Earth”). (**a**) Grid-added image; (**b**) filtered image using abwFMGFI; (**c**) filtered image using NLMF (templateWindowSize: 21, searchWindowSize: 7); (**d**) filtered image using GF (σ_x: 5, σ_y: 5); (**e**) filtered image using MF; (**f**) filtered image using BF (σ_c: 35, σ_s: 5); (**g**) filtered image using ABF (σ_s: 7, max_σ_c: 35).

**Figure 31:**
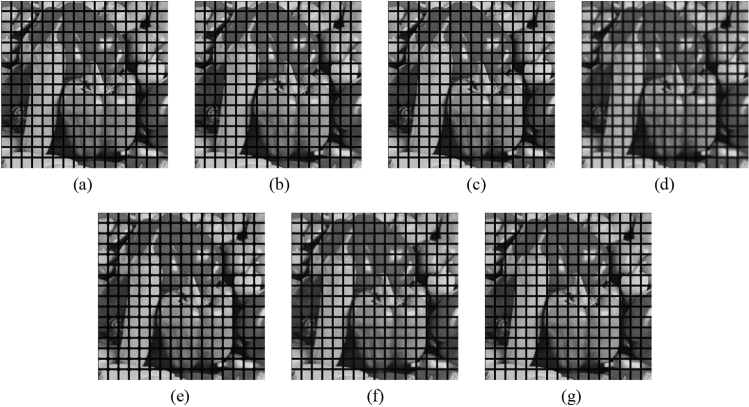
Comparison of edge preservation performance of each filter (“Pepper”). (**a**) Grid-added image; (**b**) filtered image using abwFMGFI; (**c**) filtered image using NLMF (templateWindowSize: 21, searchWindowSize: 7); (**d**) filtered image using GF (σ_x: 5, σ_y: 5); (**e**) Filtered image using MF; (**f**) filtered image using BF (σ_c: 35, σ_s: 5); (**g**) filtered image using ABF (σ_s: 7, max_σ_c: 35).

First, as before, the proposed method is compared with the NLMF, GF, MF, BF, and ABF. Figures 27, 28, 29, 30 and 31 show the grid-added images of the 12 sample images, with the respective filters applied to them. Tables 11 and 12 show the RMSE and SSIM values, respectively, for each sample image filtered using each filter. In Tables 11 and 12, the values for the filtered images with uneven and rugged edges or blurred edges are indicated in gray. The proposed method, NLMF, and ABF are confirmed to preserve the edges in visual evaluation. The GF and BF blur the edges, and the MF expands the intersections of the grid. As presented in Table 11, the NLMF and ABF show lower RMSE values than the proposed method. In addition, the NLMF has the lowest RMSE value among all the filters in this experiment. As presented in Table 12, the NLMF and ABF show higher SSIM values than the proposed method. Therefore, the edge preservation performance of the proposed method is the same as that of the NLMF and ABF in the visual evaluation, although it is inferior to the NLMF and ABF in the RMSE and the SSIM evaluation. In addition, the proposed method shows a higher edge preservation performance than the GF, MF, and BF. Furthermore, we consider that the NLMF shows the highest edge preservation performance in this experiment.

**Table 11:** Comparison of the RMSE values of each sample image filtered using each filter (regarding edge preservation performance).

Image name	abwFMGFI	NLMF	GF	MF	BF	ABF
Geometric pattern	1.854	0.452	28.367	16.228	5.381	0.830
Milkdrop	3.110	1.024	24.489	14.892	6.362	2.785
Balloon	2.015	0.706	29.317	17.064	5.404	2.554
Earth	3.204	0.471	25.544	15.445	6.421	3.991
Pepper	4.508	0.797	27.616	17.014	6.179	3.427

**Table 12:** Comparison of the SSIM values of each sample image filtered using each filter (regarding edge preservation performance).

Image name	abwFMGFI	NLMF	GF	MF	BF	ABF
Geometric pattern	0.998	0.982	0.762	0.942	0.963	0.984
Milkdrop	0.987	0.984	0.771	0.926	0.962	0.975
Balloon	0.990	0.979	0.770	0.937	0.980	0.982
Earth	0.990	0.991	0.786	0.930	0.971	0.975
Pepper	0.986	0.991	0.781	0.925	0.973	0.984

**Figure 32:**
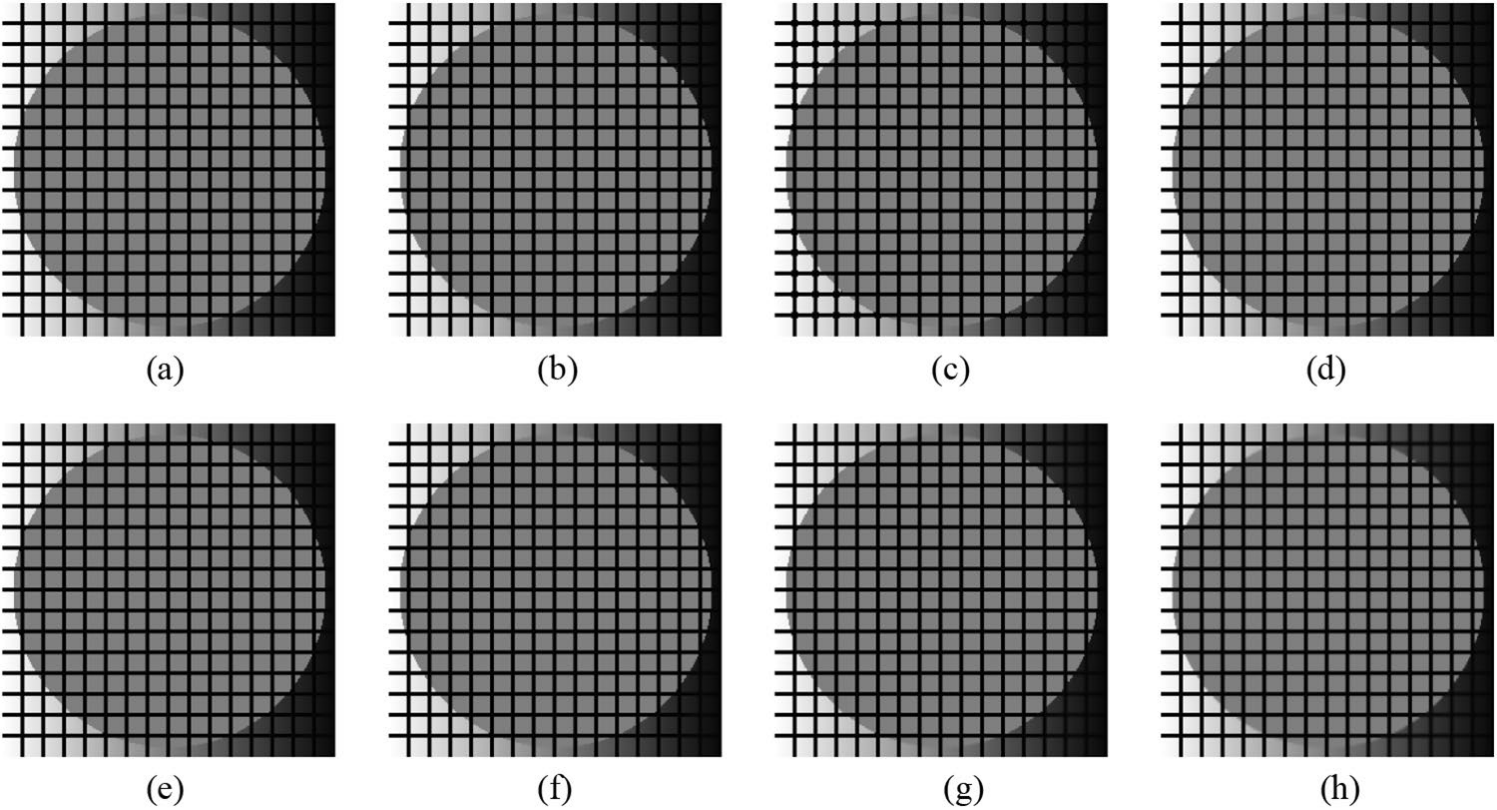
Comparison of edge preservation performance of each filter (“Geometric pattern”). (**a**) Grid-added image; (**b**) filtered image using abwFMGFI; (**c**) filtered image using FMGFI (BW = 1 level); (**d**) filtered image using FMGFI (BW = 3 level); (**e**) filtered image using FMGFI (BW = 5 level); (**f**) filtered image using FMGFI (BW = 11 level); (**g**) filtered image using FMGFI (BW = 21 level); (**h**) filtered image using FMGFI (BW = 51 level).

**Figure 33:**
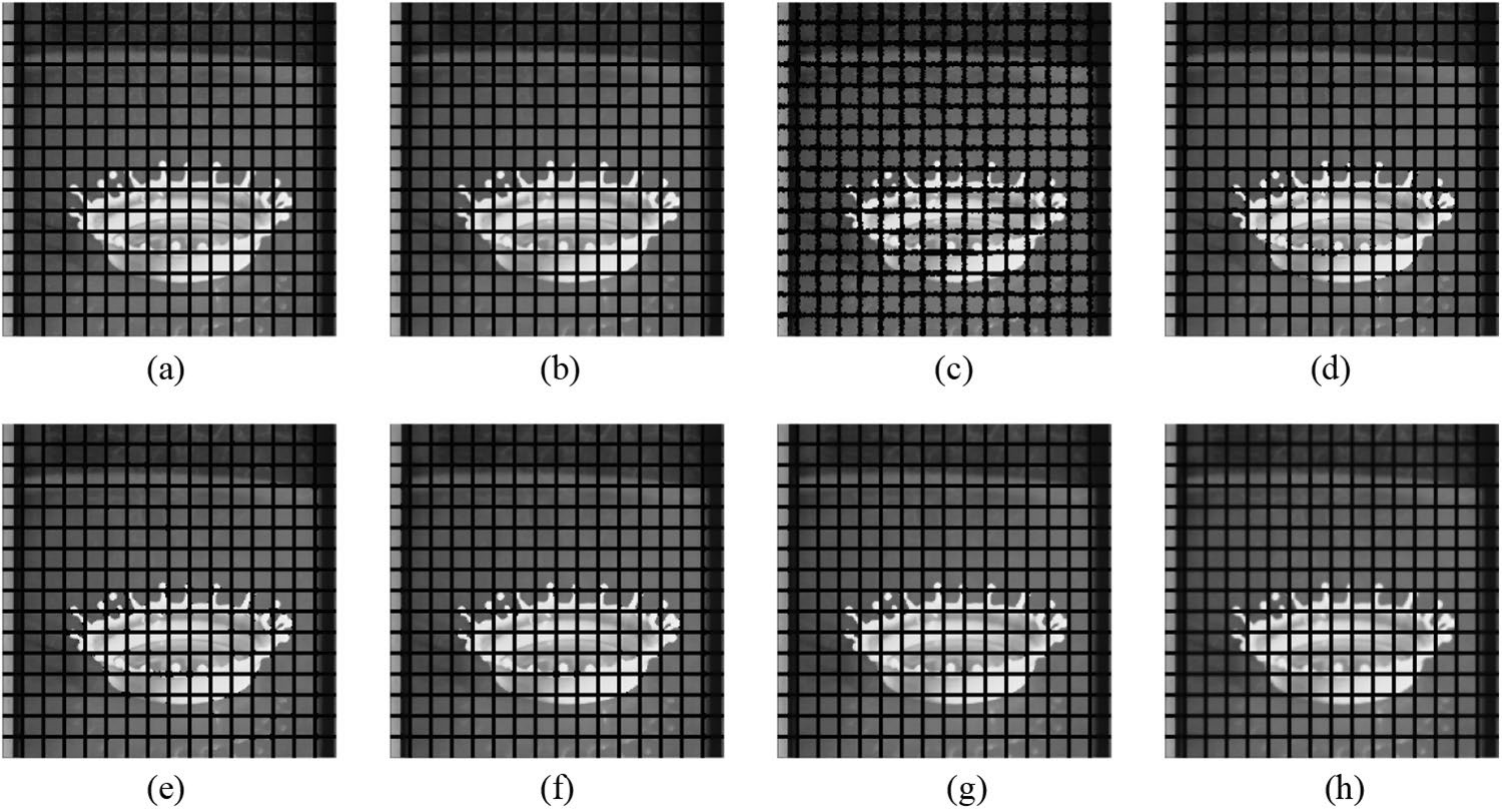
Comparison of edge preservation performance of each filter (“Milkdrop”). (**a**) Grid-added image; (**b**) filtered image using abwFMGFI; (**c**) filtered image using FMGFI (BW = 1 level); (**d**) filtered image using FMGFI (BW = 3 level); (**e**) filtered image using FMGFI (BW = 5 level); (**f**) filtered image using FMGFI (BW = 11 level); (**g**) filtered image using FMGFI (BW = 21 level); (**h**) filtered image using FMGFI (BW = 51 level).

**Figure 34:**
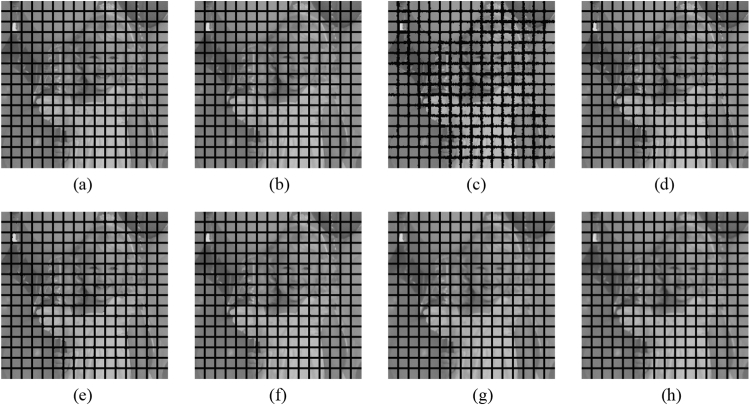
Comparison of edge preservation performance of each filter (“Balloon”). (**a**) Grid-added image; (**b**) filtered image using abwFMGFI; (**c**) filtered image using FMGFI (BW = 1 level); (**d**) filtered image using FMGFI (BW = 3 level); (**e**) filtered image using FMGFI (BW = 5 level); (**f**) filtered image using FMGFI (BW = 11 level); (**g**) filtered image using FMGFI (BW = 21 level); (**h**) filtered image using FMGFI (BW = 51 level).

**Figure 35:**
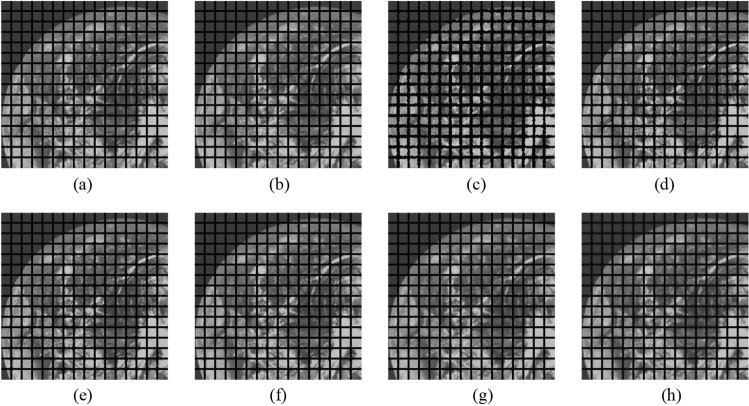
Comparison of edge preservation performance of each filter (“Earth”). (**a**) Grid-added image; (**b**) filtered image using abwFMGFI; (**c**) filtered image using FMGFI (BW = 1 level); (**d**) filtered image using FMGFI (BW = 3 level); (**e**) filtered image using FMGFI (BW = 5 level); (**f**) filtered image using FMGFI (BW = 11 level); (**g**) filtered image using FMGFI (BW = 21 level); (**h**) filtered image using FMGFI (BW = 51 level).

**Figure 36:**
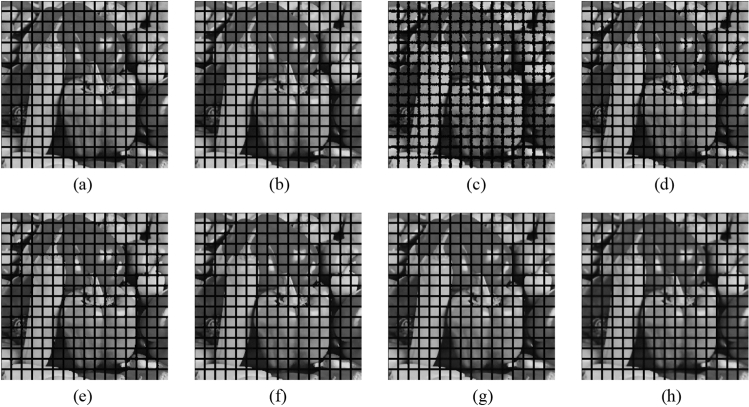
Comparison of edge preservation performance of each filter (“Pepper”). (**a**) Grid-added image; (**b**) filtered image using abwFMGFI; (**c**) filtered image using FMGFI (BW = 1 level); (**d**) filtered image using FMGFI (BW = 3 level); (**e**) filtered image using FMGFI (BW = 5 level); (**f**) filtered image using FMGFI (BW = 11 level); (**g**) filtered image using FMGFI (BW = 21 level); (**h**) filtered image using FMGFI (BW = 51 level).

Subsequently, the proposed method and the FMGFI are compared. Figures 32, 33, 34, 35 and 36 show the grid-added images of the 12 sample images, with the respective filters applied to them. Tables 13 and 14 show the RMSE and SSIM values for each sample image filtered using each filter. In Tables 13 and 14, the values for the filtered images with uneven and rugged the edges or blurred edges are indicated in gray. The proposed method and the FMGFI (basic width = 21 level) is observed to have high edge preservation performance in the visual evaluation. Further comparison shows that the edges are better preserved when using proposed method than when using the FMGFI (basic width = 21 level). The FMGFI (basic width = 1, 3, 5, 11 level) caused the edges to become uneven and rugged, the FMGFI (basic width = 51) thereby blurring the edges. As presented in Tables 13 and 14, the proposed method shows lower RMSE values and higher SSIM values than those of the FMGFI with any basic width used in this experiment. Therefore, we consider that the proposed method has a higher edge preservation performance than that of the FMGFI.

**Table 13:** Comparison of the RMSE values of each sample image filtered using the variable level of BW of FMGFI (regarding edge preservation performance).

Image name	abwFMGFI	FMGFI					
		BW = 1 level	BW = 3 level	BW = 5 level	BW = 11 level	BW = 21 level	BW = 51 level
Geometric pattern	1.854	9.076	1.955	2.059	2.046	2.241	4.141
Milkdrop	3.110	35.888	12.478	8.976	5.901	4.541	7.619
Balloon	2.015	38.903	11.736	8.016	4.215	2.606	4.266
Earth	3.204	44.378	19.653	13.009	7.675	4.574	7.166
Pepper	4.508	43.841	16.399	11.525	7.556	5.569	7.132

**Table 14:** Comparison of the SSIM values of each sample image filtered using the variable level of BW of FMGFI (regarding edge preservation performance).

Image name	abwFMGFI	FMGFI					
		BW = 1 level	BW = 3 level	BW = 5 level	BW = 11 level	BW = 21 level	BW = 51 level
Geometric pattern	0.998	0.989	0.999	0.998	0.995	0.992	0.966
Milkdrop	0.987	0.672	0.962	0.976	0.979	0.979	0.943
Balloon	0.990	0.737	0.968	0.979	0.986	0.987	0.982
Earth	0.990	0.609	0.917	0.959	0.979	0.983	0.956
Pepper	0.986	0.622	0.938	0.963	0.977	0.979	0.961

### Processing time evaluation

The high-speed processing performance of the proposed method is verified. The processing time is calculated as the average time when the filter is applied 10 times. The images used in this experiment are each of 256 × 256 pixels. The filters and filter parameters are the same as those mentioned in “Salt and pepper noise reduction experiment” Table 15 lists the specifications of the PC and programming language used.

**Table 15:** PC specification.

Filter	abwFMGFI, FMGFI	NLMF, GF, MF, BF, ABF
CPU	AMD Ryzen 9 3900 12-Core Processor 3.09 GHz	
Main memory	16 GB	
GPU	NVIDIA GeForce RTX 2060	
Programming language	MATLAB	C++
IDE	MATLAB 2020b	Visual Studio 2019, 2013
Library	–	OpenCV4.5.3, 2.4.13

Table 16 shows the processing time of the proposed method as well as those of the widely used filters, the NLMF, GF, MF, BF, and ABF.

**Table 16:** Processing time of each filter.

	abwFMGFI	NLMF	GF	MF	BF	ABF
Processing time (s)	0.359	3.328	0.02	0.005	0.004	0.028

The processing time of the proposed method is nine times faster than that of the NLMF; however, it is slower than those of the GF, MF, BF, and ABF. Precisely, the processing time of the proposed method is 0.3 s, which means that the proposed method is not sufficiently fast. Table 17 presents the processing time of the proposed method and the FMGFI. The processing time of the FMGFI increases with increase in the basic width.

**Table 17:** Processing time of each filter.

	abwFMGFI	FMGFI					
		BW = 1 level	BW = 3 level	BW = 5 level	BW = 11 level	BW = 21 level	BW = 51 level
Processing time (s)	0.359	0.145	0.179	0.201	0.269	0.386	0.800

The processing time of the proposed method is between 21 and 51 level for the basic width of the FMGFI. However, the FMGFI requires the manual setting of multiple basic widths, and the filtered images to also be manually judged. By contrast, the proposed method can automatically determine the basic width and therefore requires less time and effort than the FMGFI.

### Performance comparison of each filter

From the results obtained in the experiments discussed up to this point, the denoising, edge preservation, and high-speed processing performances of each filter are summarized in Tables 18 and 19. Table 18 shows the results of quantitative comparison of filter performance and these values are the averages of the RMSE values in Tables 3, 5, 7, 9, 11, and 13 and the processing time values in Tables 16 and 17. Table 19 shows the results of the comparison of filter performance considering quantitative and qualitative evaluations. As shown in Table 19, a greater number of circles show a higher performance. The maximum number of circles is four. In addition, X indicates that the performance is significantly low. As shown in Table 19, the proposed method shows the best denoising performance against the salt-and-pepper noise. Although the Gaussian noise is reduced when using the proposed method, its denoising performance against Gaussian noise is not as desirable as that of the ABF. On the other hand, the ABF cannot reduce the salt-and pepper noise. Although the proposed method has a high edge preservation performance, it is inferior to the NLMF. Whereas the NLMF cannot reduce the salt-and-pepper noise. Therefore, the widely used filters, the NLMF, GF, MF, BF, and ABF cannot simultaneously achieve both denoising performance against both types of noise (salt-and-pepper noise and Gaussian noise) and edge preservation performance; by contrast, the proposed method can achieve both denoising performance and edge preservation performance against these noises. Further, because the proposed method has no inferior items to the FMGFI, we can say that the proposed method is more useful than the FMGFI. The processing time of the proposed method is shorter than that of the NLMF, however, it is longer than that of the GF, MF, BF, and ABF. Therefore, we consider that the proposed method requires a faster processing speed.

**Table 18:** Quantitative comparison of filter performances.

Filter	Denoising		Edge preserve	Processing time
	Salt and pepper noise	Gaussian noise		
abwFMGFI	2.879	6.881	3.091	0.359
FMGFI	3.326	5.246	4.108	0.386
NLMF	9.942	9.961	0.722	3.328
GF	6.599	6.594	27.126	0.020
MF	3.777	5.924	16.151	0.005
BF	7.567	5.567	5.967	0.004
ABF	10.159	4.984	2.920	0.028

**Table 19:** Comparison of filter performances considering quantitative and qualitative evaluations.

Filter	Denoising		Edge preserve	Processing time
	Salt and pepper noise	Gaussian noise		
abwFMGFI	○○○○	○○	○○○	○○
FMGFI	○○○	○○○	○○	○○
NLMF	X	○○○	○○○○	X
GF	○	○	○	○○○
MF	○○	○	○	○○○○
BF	X	○	○	○○○○
ABF	X	○○○○	○○○	○○○

## Conclusions

This research was researched and developed a new denoising and edge preservation filter with aim of contributing to improvement of image quality and super-resolution technology. The findings can be summarized as follows:

First, the previous method requires human judgment for the setting of the basic width of the FMGFI, whereas in the case of the proposed method, this can be done automatically. Therefore, the proposed method successfully eliminates the need to set parameters other than the filter width, as in the MF. Therefore, the proposed filter is easier to use as compared to the FMGFI, GF, BF, ABF, and NLMF, all of which require the setting of parameters other than the filter width.

Second, the experiment results showed that the proposed method has the highest denoising performance against the salt-and-pepper noise compared to other previous filters such as the NLMF, GF, MF, BF, ABF, and FMGFI. Additionally, the denoising performance against the Gaussian noise of the proposed method is considered to be comparable to that of widely used filters such as the NLMF and ABF.

Third, from the edge preservation experiment, it is found that the edge preservation performance of the proposed method is comparable to that of the NLMF and ABF in the visual evaluation, even though it was inferior to the NLMF and ABF in the RMSE and the SSIM evaluation.

Forth, the proposed method has higher denoising and edge preservation performance than the FMGFI because it calculates the optimal basic width for each pixel, whereas the FMGFI applies the same fixed basic width for all pixels.

From these results, the abwFMGFI is expected to contribute to the improvement of image quality of images including noises. In addition, the abwFMGFI is expected to contribute to the image recognition and super-resolution technology by using it as a preprocessing. Considering further improvement, the proposed method needs to be improved because the denoising performance against the Gaussian noise is not as well as the other filters. Furthermore, the proposed method needs to be faster because a processing time of 0.3 s, as noted in the results, is not sufficiently short. Therefore, in the future, we plan to improve the performance of the proposed method against various types of noises and accelerate the processing speed.

## Supplementary Information

Supplementary Information.

## Data Availability

The datasets that support the findings of this study are available from the corresponding author on reasonable request. In addition, the datasets analyzed during this study are available in the [The USC-SIPI Image Database, https://sipi.usc.edu/database/] repository and [Standard Image Data-BAse(SIDBA), http://www.ess.ic.kanagawa-it.ac.jp/app_images_j.html] repository.
